# Turkish Adaptation, Reliability, and Validity Study of the Vaccine Acceptance Instrument

**DOI:** 10.3390/vaccines12050480

**Published:** 2024-04-29

**Authors:** Ayça Kömürlüoğlu, Esra Akaydın Gültürk, Sıddika Songül Yalçın

**Affiliations:** 1 Department of Pediatrics, Faculty of Medicine, Sivas Cumhuriyet University, Sivas 58140, Türkiye; 2 Department of Biostatistics, Faculty of Medicine, Sivas Cumhuriyet University, Sivas 58140, Türkiye; eakaydin@cumhuriyet.edu.tr; 3 Division of Social Pediatrics, Department of Pediatrics, Faculty of Medicine, Hacettepe University, Ankara 06430, Türkiye; siyalcin@hacettepe.edu.tr

**Keywords:** vaccine hesitancy, vaccine, scale validation, reliability, vaccine acceptance

## Abstract

This research study aimed to assess the reliability and validity of the Turkish version of the Vaccine Acceptance Instrument (VAI). The VAI is a 20-item Likert-type scale, with responses ranging across seven points. A systematic approach was followed to translate the scale into Turkish, involving translation, expert panel evaluation, back-translation, and pilot testing. The Vaccine Acceptance Instrument and a sociodemographic data form were used for data collection. The reliability of the scale was tested by test–retest analysis, and its internal reliability was examined by Cronbach’s alpha test. The factor structure was examined using Exploratory Factor Analysis (EFA). Confirmatory Factor Analysis (CFA) was employed to assess the scale’s fit. Overall, 229 participants were included in the study. In test–retest reliability analysis, the intraclass correlation coefficient of the scale was 0.992 (95% CI: 0.987–0.996). The Cronbach’s alpha value of the scale was 0.824. A four-factor structure was determined. The model had an acceptable fit [χ^2^/df = 380.04/164 (2,317) *p* < 0.001, CFI = 0.91, GFI = 0.90, AGFI = 0.906, NFI = 0.90, RMSEA = 0.076]. The mean total VAI score was 112.71 ± 17.02. The low education level of the mother, being a housewife, and parents not having the COVID-19 vaccine were statistically significantly associated with a low scale score and low vaccine acceptance (*p* < 0.05). The Turkish adaptation of the VAI demonstrated satisfactory levels of validity and reliability following rigorous testing.

## 1. Introduction

Vaccination is the most successful, safest, and cost-effective approach to protecting children’s health, preventing infectious diseases, and saving the lives of millions of children every year. With vaccination programs, mortality and morbidity of infectious diseases have decreased; smallpox has been eradicated all over the world, and polio has been eradicated in many countries, including our country [1]. The decline in vaccination rates as a result of vaccine refusal (VR) and vaccine hesitancy (VH), which is actually as old as the history of vaccines but has recently increased in our country and all over the world, is remarkable due to the emergence of vaccine-preventable infectious disease outbreaks and the danger of decreasing social immunity. Vaccine refusal and VH are two of the most important problems of our age [2]. Vaccine refusal cases are increasing in our country [3].

According to the World Health Organization (WHO), vaccine hesitancy is defined as “a reluctance or refusal to accept vaccines even when vaccination services are readily available”, while vaccine refusal refers to “the act of not vaccinating children due to a decision to decline all vaccines”. In essence, vaccine hesitancy denotes the hesitance or reluctance to accept vaccination services despite their availability [4]. In 2012, the ‘Vaccine Hesitancy Working Group’ was established within the Strategic Advisory Group of Experts on Immunization (SAGE) by WHO. First of all, the concepts of vaccine refusal and vaccine hesitancy were defined, and then the “Vaccine Hesitancy Model” was created, which takes place in three main areas (contextual influences, individual and group influences, and vaccine and vaccination-specific issues) [5]. Social media, vaccine lobbies, influential leaders, religious, cultural, geographical, social, political, and economic factors, perceptions about the pharmaceutical industry, effects of the social environment, experiences regarding the vaccine, beliefs and attitudes about health, knowledge and awareness, perceived risks related to vaccination, social norms regarding vaccination, trust in the healthcare system and healthcare professionals, benefit/cost ratio, implementation and management of the vaccination program, attitudes of healthcare professionals, and the strength of their recommendations are the main reasons underlying VH [2,5,6,7,8]. Psychological factors such as conspiratorial, religious, paranoid beliefs, and moral purity have also been associated with VR [9].

With the definition of VH and the identification of its components, the idea of developing measurement tools to measure VH has emerged. Identifying parents with VH and the underlying reasons will also help to develop global solutions to increase childhood vaccination rates, so universal scales measuring vaccine acceptance and vaccine hesitancy have begun to be developed [10]. For researchers, in order to be able to conduct studies in their own culture and language, and to work with different patient groups, they need validated and reliable measurement tools in their own language. There are various scales in the literature that evaluate parents’ vaccine hesitancy [11]. The most widely used worldwide of these is Opel et al.’s, which was developed in 2011, ‘Parental Attitude Scale (PACV) on Childhood Vaccinations’. High scores from this 18-item scale indicate opposition to vaccination, while low scores indicate vaccine acceptance [12]. Gilkey et al. developed the 8-item ‘Vaccine Confidence Scale’ (VCS) [13]. Larson et al. aimed to evaluate parents who have VH with the 10-item ‘Vaccine Hesitancy Scale’ [14]. Wallace et al. aimed to determine parents’ attitudes towards vaccines with the 11-item ‘Vaccine Attitude Scale’ [15]. Accurately measuring VH, and vaccine acceptance is crucial for enhancing vaccination rates, formulating targeted vaccination policies, and implementing evidence-based interventions. This necessity becomes particularly pronounced in contemporary contexts where vaccine misinformation can exacerbate public apprehension and politicize vaccination strategies. Sarathchandra et al. have introduced a novel instrument, the “Vaccine Acceptance Instrument (VAI)”, designed to complement existing measures and comprehensively evaluate parental attitudes towards vaccination [16]. Derived from a meticulous review of the literature and informed by interdisciplinary insights from public health, humanities, and social sciences, this scale, alongside its five subscales, exhibits robust psychometric properties, including high Cronbach’s alpha coefficients and factor analysis results. Notably, there exists a gap in the literature regarding the validation and reliability of this scale within the Turkish context. Hence, the primary objective of this study is to adapt the “VAI” developed by Sarathchandra et al. into Turkish and assess its validity and reliability.

The study is anchored in the social cognitive theory, which underscores the dynamic interplay between individual cognition, behavior, and environmental influences in shaping health-related decision-making [17]. Vaccine acceptance can be analyzed via the lens of social cognitive theory, which plays a crucial role in comprehending and predicting behavior, particularly in health contexts. These theories, grounded in reasoned action, suggest that individuals’ engagement in behavior is shaped by their beliefs and judgments about its future consequences, which can be influenced by information-provision strategies in behavioral interventions. Interventions informed by social cognitive theories have proven effective in altering behavior, including vaccine intentions amid the COVID-19 pandemic [18,19]. However, challenges persist in applying these theories, such as a focus on intentions over actual behavior, limited longitudinal research, and an over-reliance on cross-sectional designs, limiting understanding of causality and change over time. Moreover, there is a need to expand measures beyond broad constructs of social cognition to include specific beliefs relevant to preventive behaviors. Alternatively, the Health Belief Model (HBM) offers insights into motivations for health-related behaviors. The HBM provides a theoretical lens through which to understand the cognitive processes underlying vaccination attitudes and behaviors, emphasizing perceived susceptibility, severity, benefits, and barriers as key determinants [20]. Studies by Cheney and John [21] revealed significant differences in health beliefs between vaccine-reluctant and vaccine-accepting individuals. Given that health beliefs may vary between countries and communities, it is essential to scrutinize the validity and reliability of scales developed to measure these beliefs across diverse populations [22]. Specifically, we anticipate that the scale will effectively capture the multifaceted dimensions of vaccine acceptance and hesitancy among Turkish parents, thereby facilitating targeted interventions aimed at addressing underlying determinants of vaccination behavior.

## 2. Materials and Methods

### 2.1. Study Sample

The data for this methodological research was collected between March 2022 and May 2023. Parents who applied to the outpatient clinics of Sivas Cumhuriyet University Department of Pediatrics for healthy child follow-up, and who had children between the ages of 0–16, who did not have chronic diseases, and who agreed to participate in the study were included in the study. The criteria for inclusion in the study are that the parent has no mental disability, can read and write, and speaks fluent Turkish. One of the parents was involved in the study. Other family members were not included. Illiterate parents were not included either. All participants were informed about the study and the scale beforehand, and their consent was obtained.

### 2.2. Study Design

In calculating the sample size for validity and reliability studies, the recommended number of participants is to include 5–10 times the number of items in the scale [23]. A sample size of 10 cases per item was calculated. To account for potential incomplete or erroneous questionnaire completions, it was planned to reach 20% more respondents. The data was collected using the pen-and-paper interview method under the supervision of one of the researchers. 

### 2.3. Participation Rate

During the data collection period, the survey was given to 240 participants. Six parents refused to participate and declared that they did not have enough time to participate. Five surveys were excluded from the study due to incompleteness. A total of 229 participants participated in the study. The participation rate was 95.4% (229/240). 

### 2.4. Questionnaire

In collecting data, the sociodemographic information form created by the researchers was used, and the Vaccine Acceptance Instrument (the Turkish version of the VAI) was applied.

The scale we used for the study, “VAI”, consists of twenty items and five subscales. There is a 7-point Likert-type scoring scale (strongly disagree 1 point- strongly agree 7 points). Reverse coding is carried out for the items 2-3-4-5-6-11-12-13-14-16-17-18. The total score is calculated by the sum of the scores given to the items. This scale was developed by Sarathchandra D, Navin MC, Largent MA, McCright MA, piloted in 2015, applied to 250 American adults in 2016, and the results were evaluated and published in 2018 [16]. This scale measures five key facets of vaccine acceptance: (1) *perceived safety of vaccines (items 1–4)*, (2) *perceived effectiveness and necessity of vaccines (items 5–8)*, (3) *acceptance of the selection and scheduling of vaccines (items 9–12)*, (4) *positive values and affect towards vaccines (items 13–16)*, and (5) *perceived legitimacy of authorities to require vaccinations (items 17–20)*. The scale does not have a cut-off point. A high scale score indicates high vaccine acceptance. Cronbach’s alpha value for the whole scale was 0.96. Cronbach’s alpha value of the subscales is between 0.81 and 0.91. The scale also has a short form consisting of 10 items.

### 2.5. Process

**Figure 1:**
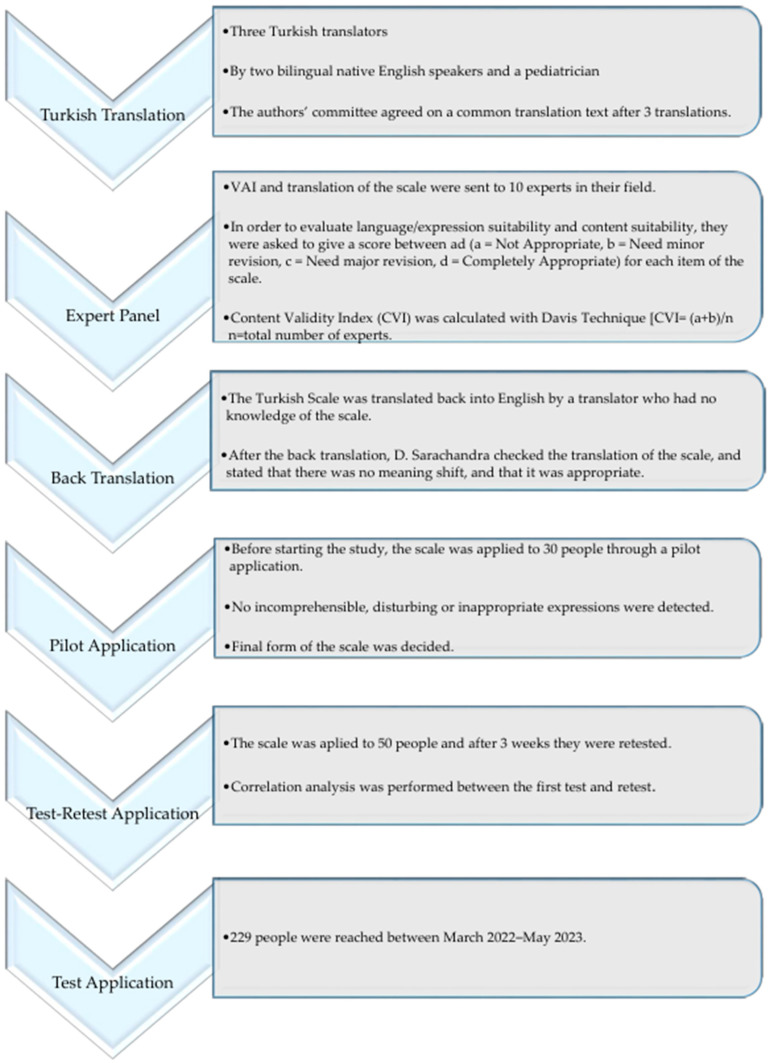
The research implemented stages of intercultural adaptation to ensure the validity of the translated scale.

We received permission via e-mail from D. Sarathchandra, one of the original authors, to use the VAI. The World Health Organization’s intercultural adaptation guidelines [24] and the literature review’s suggestions were followed for completing the scale’s adaptation steps. Three bilingual Turkish translators were engaged in translating the scale into Turkish. Subsequently, the committee of authors reached a consensus on a unified translation of the text. Ten specialists were sent the translated scale to evaluate. The opinions given by the experts were evaluated using the Davis technique [25], and the content validity index (CVI) was calculated. No items were removed from the scale in line with expert evaluations. After translating the Turkish scale back into English, confirmation was sought from D. Sarathchandra via email to ensure accuracy. A pilot study using the Turkish version of the scale involved 30 participants. The final version of the scale was then tested and re-tested with 50 people. Test–retest correlation analysis was investigated. After that, the main study involved administering the data collection form and VAI to 229 participants. Figure 1 illustrates the steps of intercultural adaptation that were implemented.

### 2.6. Ethical Aspect of the Research

This study was approved by the Sivas Cumhuriyet University Non-Interventional Clinical Research Ethics Committee (with the 13 January 2022 date and 2022-01/15 number) in accordance with the Declaration of Helsinki. Prior to the study, the participants were informed about the study and gave their consent.

### 2.7. Data Analyses

Data analysis was conducted using IBM Statistical Package for Social Sciences (SPSS) version 23.0 (SPSS Inc., Chicago, IL, USA) and Lisrel 8.8 (Scientific Software International, Inc., Chapel Hill, NC, USA). The normality of numerical values was assessed using the Shapiro–Wilk test. Descriptive statistics were computed for sociodemographic variables and scale items. The comparison of normally distributed numerical data between two categorical variables was conducted using Student’s *t*-test, while the ANOVA test was employed for comparisons across more than two categorical variables. The threshold for statistical significance was established at *p* < 0.05, with a confidence interval (CI) of 95%.

An investigation into test–retest reliability was conducted to assess intratester reliability using intraclass correlation. A two-way mixed model with absolute agreement was selected. In the literature, it is stated that this value should be 0.70 and above [26]. Additionally, internal consistency was assessed using Cronbach’s alpha coefficient. A Cronbach’s alpha value above 0.80 is an indicator of strong reliability [27]. As the second reliability analysis, a split-half reliability analysis was performed. Studies show that this Spearman–Brown value should be the same or higher than Cronbach’s alpha value [28]. The scale’s suitability for factor analysis was assessed via the Kaiser–Meyer–Olkin (KMO) measure and Bartlett’s test of sphericity. A KMO value exceeding 0.60 and a significant result in Bartlett’s test indicate the appropriateness of the data for factor analysis [23,24,25,26,27,28,29]. Exploratory factor analysis (EFA) was conducted to explore the interrelationships among items from the questionnaire and to determine the number of factors. Exploratory factor analysis was performed on the 20 vaccine acceptance items using Principal Components Analysis (PCA) with Promax rotation and Kaiser normalization. We used a factor loading cut-off of >0.3 to determine which items belonged to the identified factors in EFA. An item was categorized under the factor in which it loaded the highest when it loaded on multiple factors [30]. 

Confirmatory factor analysis (CFA) was employed to test the model derived from EFA by specifying factors as either correlated or uncorrelated a priori. Confirmatory factor analysis results were reported with χ^2^/df, comparative fit index (CFI), goodness-of-fit index (GFI), normed fit index (NFI), adjusted goodness-of-fit index (AGFI), and root-mean-square error of approximate (RMSEA) values. χ^2^ is not a statistic evaluated on its own but is evaluated in proportion to the degrees of freedom. A value below 2 in this ratio indicates a good fit, and a value below 3 indicates an acceptable fit. However, among other indices used to evaluate compliance, RMSEA must be 0.08 and below, and CFI, GFI, AGFI, and NFI values must be 0.90 and above [29,31]. 

## 3. Results

### 3.1. Descriptive Characteristics of the Participants

The study comprised 229 parents with healthy children aged between 0 and 16 years. Among the survey respondents, 86.9% (*n* = 199) were mothers. Seventy-four percent of the mothers had a high school education or higher, and 54.1% were housewives. In total, 80.3% of the fathers were university graduates and 22.3% were working in the education and health sectors. The parents’ average number of children was 2 (median) years, and the average age of their children was 6.96 ± 4.38 (mean ± SD) years. The income level of 26.2% of the families was at the minimum wage or below. A total of 96.9% of the children were fully vaccinated in accordance with the vaccination schedule. Additionally, 94.3% of mothers and 93.9% of fathers had received at least two doses of the COVID-19 vaccine. 

The average VAI score of the participants was 112.71 ± 17.02. The sociodemographic characteristics of the parents and their children participating in the study, the comparison of the participants’ VAI scores subgroup scores with their demographic data is shown in Table 1. The VAI score of those with maternal education levels below high school was statistically significantly lower than that of those with high school and above, and the VAI score of housewife mothers was statistically significantly lower than that of working mothers (*p* = 0.043 and *p* = 0.005). Parents with high monthly income had statistically significantly higher scale scores than those with low and medium monthly income (*p* < 0.05). The scale score of parents who received at least 2 doses of the COVID-19 vaccine was higher than those who did not receive it, and this difference was statistically significant in mothers (*p* = 0.03). The scale score of parents whose children were incompletely vaccinated/unvaccinated was lower than those whose children were fully vaccinated, but the difference was not statistically significant. No significant correlation was found between the VAI score and sociodemographic characteristics. 

### 3.2. Validation and Consistency Analyses of the VAI

**Figure 2:**
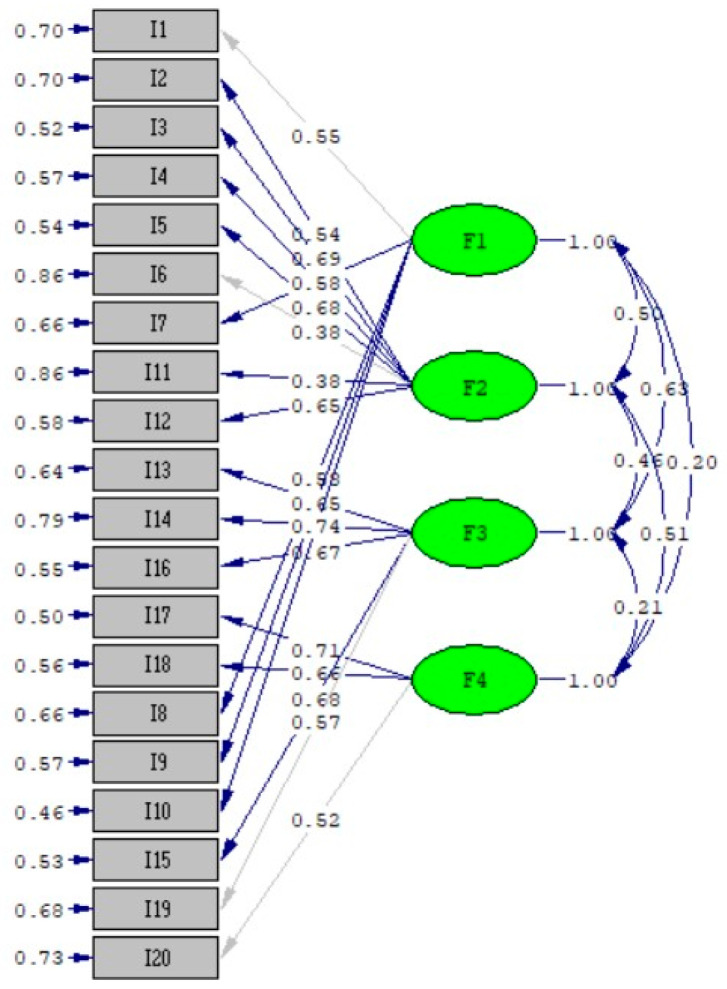
Confirmatory factor analysis model of the VAI.

The content validity indexes of the scale items ranged from 0.86 to 1.00 (Table 2). CVI above 0.80 indicates good content validity [29]. The KMO coefficient was found to be 0.787, and Bartlett’s sphericity test results were significant (χ^2^(190): 1358.608, *p* < 0.01). The sample size was sufficient, and correlations between items were large enough for EFA. With EFA, it was determined that there were four factors with eigenvalues above 1 on the Turkish scale, and the contribution of these factors to the total variance was 51.9%. Confidence in vaccines, the vaccination schedule, and the belief that vaccines prevent serious diseases constitute the positive vaccine perceptions subscale (F1: items 1-7-8-9-10). Items containing the thoughts that vaccines contain harmful substances, that vaccines are unnecessary, and that too many and untimely vaccines are given to children constitute the negative vaccine perceptions subscale (F2: items 2-3-4-5-6-11-12). Items addressing the moral, humanitarian, and behavioral dimensions of vaccination constitute the social norms of the vaccines subscale (F3: items 13-14-15-16-19), while items 17-18-20 constitute the perception of voluntariness and obligation in the vaccines subscale (F4). Factor loadings for F1 ranged from 0.506 to 0.766; for F2, 0.325 to 0.765; for F3, 0.587 to 0.735; and for F4, 0.711 to 0.816. EFA results of the VAI are given in Table 3. The factor loads in Table 3 are expressed in accordance with the 4-factor model’s pattern matrix outcome in Figure 2. Confirmatory factor analysis showed the ratio of the χ^2^ value to the obtained degrees of freedom was 2.317 (*p* < 0.001). Among other fit indices, RMSEA was found to be 0.076, GFI was 0.90, AGFI was 0.906, CFI was 0.910, and NFI was 0.90 (Table 4). The fit indices of the scale were at an acceptable level. We can say that the Vaccine Acceptance Scale Turkish form is valid.

The internal consistency of the Turkish version of the VAI was assessed using Cronbach’s alpha coefficient, and a value of 0.824 was obtained. Additionally, the Cronbach’s alpha coefficients for its subscales ranged from 0.662 to 0.754. Item-total correlation coefficients ranged from 0.241 to 0.615, with only one value falling below 0.30. However, this item was retained in the scale as its removal had no significant impact on Cronbach’s alpha score. Split-half reliability analysis was performed, and Spearman–Brown coefficient was found to be 0.879. It was higher than Cronbach’s alpha value of the full scale. Furthermore, reliability was evaluated via the test–retest method, revealing a high intraclass correlation coefficient (ICC) of 0.992 (95%CI: 0.987–0.996) between the initial and final measurements (Table 5). These values show that the Vaccine Acceptance Instrument Turkish form is reliable. 

## 4. Discussion

Understanding parents’ attitudes and opinions towards vaccination is essential for the success of immunization programs. Utilizing validated and reliable scales is imperative for effectively measuring vaccine acceptance and hesitancy. Via this investigation, the VAI was successfully adapted to Turkish, administered to 229 parents, and deemed valid and reliable. Notably, the Turkish version retained the original scale’s 20 items. Evaluation of the CVI indicated high values ranging from 0.86 to 1.00, suggesting that the scale items effectively capture the intended constructs. However, distinct from the original scale, our analysis revealed a four-factor structure for the Turkish version. These factors accounted for 51.9% of the total variance, contrasting with the three factors identified in the original study by Sarathchandra et al. [16], which explained 59.96% of the variance. It is noteworthy that our factor analysis results align well within the acceptable range of explained variance between 40% and 60%, signifying adequate measurement of the underlying constructs [32]. 

In our study, the 4-factor structure scale was named Factor 1: Positive Vaccine Perceptions, Factor 2: Negative Vaccine Perceptions, Factor 3: Societal Norms of Vaccines, Factor 4: Voluntariness and Obligation Perception in Vaccination. The item-total correlation of the scale was between 0.241 and 0.615. It is stated that the item-total correlation coefficient should not be lower than 0.30 [29,30]. One value fell below 0.30; however, upon removal of this item, Cronbach’s alpha remained unchanged. Therefore, it was decided to keep this item on the scale. The Hotelling T^2^ value calculated to determine whether the scale items are perceived similarly by the participants [33] is significant (*p* < 0.001). The Turkish form of the VAI can be considered a powerful and original scale consisting of questions with a homogeneous structure.

In our study, the scale’s Cronbach’s alpha coefficient was 0.824, and the Cronbach’s alpha values of the sub-dimensions were above 0.66. In existing literature, a Cronbach’s alpha value of 0.60 or higher is considered acceptable [29]. In Split-half reliability analysis, the Spearman–Brown coefficient was higher than Cronbach’s alpha value. These values show that the Turkish form of the VAI is reliable. Sarathchandra et al. determined the Cronbach’s alpha coefficient of the VAI full scale to be 0.96 and stated that the Cronbach’s alpha coefficients calculated for the scale sub-dimensions ranged between 0.81 and 0.91 [16].

The test–retest method was used to measure the reliability of the scale [34]. In this study, the test was administered again to 50 participants three weeks after first completion. The intraclass correlation of the scale was 0.992 (95% confidence interval: 0.987–0.996). Intraclass correlations of subgroups vary between 0.945 and 0.986. A robust and statistically significant positive correlation was observed between the two sessions (*p* < 0.05). 

The VAI mean score of the parents was 112.71 ± 17.02. The study did not calculate a specific cut-off value. A cut-off value for the scale can be determined via further studies with risk group individuals (autism, etc.) in terms of vaccine hesitancy and vaccine refusal. There is no age limit for the original scale. It can be said that the Turkish scale can be applied to all parents without any age limit for their children.

Following factor and reliability analyses, a 20-item scale comprising four factors and four subscales, exhibiting a range of fit index values within acceptable and ideal thresholds, emerged. A high scale score indicates high vaccine acceptance. Remarkably, mothers with education levels below high school, unemployed individuals, and parents who had not received the COVID-19 vaccine demonstrated notably lower scale scores, indicative of low vaccine acceptance and heightened vaccine hesitancy. This observation aligns with a contentious topic in the literature. Indeed, analogous to our findings, previous studies have reported associations between lower parental education levels, socioeconomic status, and vaccine opposition or refusal [35,36,37], and there are also studies with the opposite view [38,39].

During the COVID-19 pandemic, there has been a noted increase in anti-vaccine movements. Social, economic, political, and psychosocial factors also contributed to VH and VR during the pandemic [9]. In one of the studies investigating the psychosocial causes of COVID-19 vaccine hesitancy, cognitive attitude, descriptive norms, and perceived behavioral control significantly predicted parental vaccination intention [40]. In another study, political conservatism, reactivity, religiosity, and low socioeconomic level all significantly contribute to greater vaccine hesitancy [41]. Soysal et al. [42] observed that the refusal of childhood vaccines rose concurrently with the rejection of COVID-19 vaccines. Similarly, our study revealed lower scale scores among individuals who had not received the COVID-19 vaccine compared to those who had. Upon closer examination of subscale scores, it became evident that the negative vaccine perceptions subscale score was notably lower among parents who had not received the COVID-19 vaccine, particularly in mothers, with statistical significance (*p* < 0.05). These findings are consistent with existing literature. Furthermore, our results indicated that the perception of voluntariness and obligation in the vaccination subscale score was significantly lower among individuals with incompletely vaccinated or unvaccinated children compared to those with fully vaccinated children (*p* < 0.05). This finding holds significance as it reflects the stance of vaccine-hesitant individuals regarding the voluntariness or compulsion of vaccination. Determining the scale and subscale cut-off values could be achieved via further research involving larger populations and a substantial number of vaccine refusal cases. 

In our study, VAI was used to evaluate parents’ acceptance of vaccination. There are also studies evaluating vaccine acceptance in different groups other than parents using VAI or its subgroups in the literature. Führer et al. used the short form of VAI when evaluating COVID-19 vaccine acceptance of German migrants [43]. They found that vaccine acceptance was higher in vaccinated participants. There are also studies conducted on healthcare personnel, firefighters, and general populations [44,45,46]. Pivetti et al. [47] used the VAI short form translated into Italian in their study involving 590 participants to evaluate COVID-related conspiracy beliefs and COVID-19 vaccine acceptance. They found that conspiracy beliefs negatively predicted general attitudes towards vaccines.

In their investigation, Sarathchandra et al. [16] conducted a comprehensive examination, concurrently assessing scientific literacy, conspiratorial thinking, political ideology, religiosity, trust in scientists, and vaccine acceptance. This was accomplished using Drummond and Fischhoff’s 11-item Scientific Reasoning Scale [48], Lewandowsky et al.’s 12-item Conspiracist Ideation Scale [49], and the 15-item Trust in Biologists Scale [16]. Notably, trust in biologists exhibited a moderately strong positive influence, while conspiratorial ideation demonstrated a moderately strong negative impact on all dimensions of vaccine acceptance and the overall scale. These findings were consistent with existing literature. In contrast, our study focused solely on the Turkish validation and reliability assessment of the VAI without incorporating additional scales. However, we extended our analysis to include comparisons between sociodemographic characteristics and vaccination status with the VAI score. In future inquiries, researchers may delve into further factors that contribute to vaccine hesitancy and refusal. Evaluating Vaccine Acceptance (VA) and Vaccine Hesitancy (VH) requires a holistic approach, considering numerous interconnected factors that reach beyond individual beliefs or attitudes towards vaccines. This entails understanding broader socio-cultural, economic, and political contexts, as well as factors like trust in healthcare systems and community engagement, all of which shape individuals’ perspectives and behaviors concerning vaccination [7].

### Strengths and Limitations

Our study is subject to several limitations. Firstly, the sample used in this study was not representative of the entire Turkish population, as data were collected from a single center. Consequently, the findings may not fully reflect the diverse perspectives on vaccine acceptance and hesitancy across different regions of Türkiye [3]. Future research endeavors employing the VAI on a national scale, encompassing various regions and demographics, could offer valuable insights into potential regional disparities in vaccine attitudes.

To our knowledge, this study represents the first Turkish validity and reliability assessment of the long form of VAI, marking a significant contribution to the field. Our study is one of the few studies conducted in a language other than the original version of the VAI long form. This pioneering effort expands the accessibility of the scale to non-English speaking populations, thereby enhancing its utility and relevance on a global scale. Consequently, our findings provide a valuable foundation for further research and discourse on vaccine acceptance and hesitancy within the Turkish context.

## 5. Conclusions

In conclusion, the successful adaptation of the Vaccine Acceptance Instrument (VAI) to Turkish, demonstrated via rigorous reliability and validity assessments, establishes its utility as a valuable tool for identifying vaccine hesitancy. The implementation of this scale is anticipated to significantly contribute to understanding parental vaccination attitudes in our country. Moreover, healthcare professionals, including doctors, nurses, and researchers across various fields, can confidently utilize the VAI to gather pertinent data from parents, thereby facilitating the planning of educational initiatives and the development of strategic interventions to address vaccine hesitancy. Ultimately, the insights gleaned from utilizing the VAI have the potential to inform health policies aimed at promoting vaccination acceptance. Looking ahead, further research can expand upon our findings by exploring vaccine acceptance levels in diverse populations and investigating its relationship with various influencing factors, paving the way for more comprehensive studies in the field of vaccination acceptance and hesitancy.

## Figures and Tables

**Table 1:** Sociodemographic characteristics of the participants, scale, and subscale scores.

	*n* (%)	VAI Score (Mean ± SD)	*p* Value	F1 Subscale Score (Mean ± SD)	F2 Subscale Score (Mean ± SD)	F3 Subscale Score (Mean ± SD)	F4 Subscale Score (Mean ± SD)
Parent			0.73				
Mother	199 (86.9%)	112.89 ± 16.19		30.32 ± 5.21	35.6 ± 8.54	32.13 ± 4.38	14.83 ± 5.25
Father	30 (13.1%)	111.46 ± 22.05		29.86 ± 6.06	35.66 ± 10.36	31.66 ± 5.79	14.26 ± 5.21
Child age (year)	(6.96 ± 4.38)		0.72				
≤5 years	104 (45.4%)	113.14 ± 17.63		30.33 ± 4.67	35.83 ± 9.34	32.03 ± 4.36	14.93 ± 5.40
6 years and older	125 (54.6%)	112.35 ± 16.55		30.2 ± 5.82	35.42 ± 8.31	32.1 ± 4.76	14.61 ± 5.11
Child gender			0.63				
Male	103 (45%)	112.12 ± 17.77		30.07 ± 5.13	35.31 ± 9	31.55 ± 5.05	15.18 ± 5.02
Female	126 (55%)	113.19 ± 16.44		30.42 ± 5.49	35.85 ± 8.61	32.5 ± 4.11	14.41 ± 5.40
Mother’s age (years)	(36.12 ± 6.65)		0.24				
<35	100 (43.7%)	111.21 ± 16.8		30.13 ± 4.89	35.06 ± 8.75	31.75 ± 4.12	14.27 ± 5.69
≥35	129 (56.3%)	113.87 ± 17.18		30.37 ± 5.65	36.03 ± 8.80	32.32 ± 4.9	15.13 ± 4.85
Father’s age (years)	(39.62 ± 6.78)		0.66				
<35	57 (24.9%)	111.8 ± 17.84		30.14 ± 5.43	35.31 ± 9.18	31.75 ± 4.18	14.49 ± 5.59
≥35	172 (75.1%)	113.01 ± 16.78		30.3 ± 5.30	35.7 ± 8.66	32.18 ± 4.70	14.81 ± 5.13
Family type			0.78				
Nuclear	220 (96.1%)	112.7 ± 17.27		30.21 ± 5.38	35.63 ± 8.89	32.01 ± 4.64	14.83 ± 5.21
Extended/broken family	9 (3.9%)	112.88 ± 9.94		31.44 ± 3.74	35.11 ± 5.55	33.44 ± 2.18	12.88 ± 5.79
Parent education (Mother)							
<High school	59 (25.8%)	108.86 ± 15.16	0.043	28.93 ± 6.61	35.11 ± 8.25	30.86 ± 5.32 *	13.94 ± 5.32
≥High school and higher	170 (74.2%)	114.04 ± 17.46		30.72 ± 4.72	35.78 ± 8.97	32.49 ± 4.22 *	15.04 ± 5.20
Parent education (Father)			0.14				
<High school	45 (19.7%)	109.35 ± 16.40		29.73 ± 6.26	35.11 ± 8.22	31.08 ± 5.36	13.42 ± 5.70
≥High school and higher	184 (80.3%)	113.53 ± 17.11		30.39 ± 5.08	35.73 ± 8.92	32.31 ± 4.35	15.08 ± 5.08
Household income			0.003				
Low income	60 (26.2%)	111.63 ± 15.55		30.11 ± 6.09	36.05 ± 8.24	31.06 ± 5.58	14.4 ± 5.38
Moderate income	73 (31.9%)	108.08 ± 16.35		29.83 ± 5.56	33.05 ± 8.74	31.82 ± 4.04	13.36 ± 5.26
High income	96 (41.9%)	116.90 ± 17.54		30.68 ± 4.6	37.28 ± 8.76	32.89 ± 4.15	16.04 ± 4.87
Living in							
Center	190 (83%)	112.85 ± 17.22	0.77	30.53 ± 5.08	35.42 ± 8.82	31.97 ± 4.73	14.92 ± 5.1
Others	39 (17%)	112.02 ± 16.2		28.97 ± 6.25	36.53 ± 8.61	32.56 ± 3.74	13.94 ± 5.87
Mother’s employment			0.005				
Housewife	124 (54.1%)	109.83 ± 15.54		29.98 ± 5.86	34.63 ± 8.02	31.48 ± 4.68 *	13.73 ± 5.25 *
Employed	105 (45.9%)	116.1 ± 18.1		30.6 ± 4.6	36.76 ± 9.5	32.77 ± 4.37 *	15.97 ± 4.98 *
Father’s employment			0.001				
Unemployed	2 (0.9%)	132.5 ± 10.6		30.5 ± 6.36	47 ± 2.82	34.5 ± 0.7	20.5 ± 0.7
Education sector	22 (9.6%)	115.13 ± 16.14		31.72 ± 3.41	35.68 ± 9.38	33.27 ± 3.10	14.45 ± 4.65
Health sector	29 (12.7%)	123.13 ± 14.89		31.24 ± 3.44	40.89 ± 7.83	33.31 ± 3.43	17.68 ± 4.24
Others	176 (76.9%)	110.46 ± 16.79		29.92 ± 5.73	34.60 ± 8.53	31.69 ± 4.86	14.25 ± 5.31
Number of children in the household			0.47				
1	51 (22.3%)	111.13 ± 18.39		30.8 ± 3.81	34.17 ± 10.11	32.19 ± 4.53	13.96 ± 5.47
2–3	149 (65.1%)	113.71 ± 16.48		30.07 ± 5.69	36.07 ± 8.14	32.22 ± 4.31	15.33 ± 5.06
≥4	29 (12.7%)	110.34 ± 17.41		30.31 ± 5.7	35.75 ± 9.44	31.06 ± 5.85	13.20 ± 5.42
İmmunization status of the child			0.11				
Fully vaccinated	222 (96.9%)	113.02 ± 16.73		30.26 ± 5.35	35.74 ± 8.68	32.14 ± 4.49	14.86 ± 5.26 *
incompletely vaccinated/unvaccinated	7 (3.1%)	102.71 ± 24.12		30.28 ± 4.82	31.42 ± 11.45	29.71 ± 6.84	11.28 ± 2.69 *
Mother’s COVID-19 vaccination status			0.03				
At least 2 doses given	216 (94.3%)	113.65 ± 16.04		30.5 ± 5.05	35.93 ± 8.56 *	32.36 ± 4.03	14.85 ± 5.26
Unvaccinated	13 (5.7%)	97 ± 24.73		26.38 ± 7.93	30.15 ± 10.8 *	27.3 ± 9.03	13.15 ± 4.82
Father’s COVID-19 vaccination status			0.09				
At least 2 doses given	215 (93.9%)	113.49 ± 15.97		30.45 ± 5.06	35.87 ± 8.53	32.34 ± 4.03	14.82 ± 5.25
Unvaccinated	14 (6.1%)	100.64 ± 26.75		27.42 ± 8.12	31.57 ± 11.56	27.85 ± 8.91	13.78 ± 5.1
Mother’s vaccination in the last 5 years,			0.66				
Yes	110 (48%)	113.20 ± 16.26		30.09 ± 5.10	35.99 ± 8.85	32.3 ± 3.91	14.8 ± 5.43
No	119 (52%)	112.26 ± 17.75		30.42 ± 5.53	35.26 ± 8.72	31.85 ± 5.12	14.71 ± 5.07
Father’s vaccination in the last 5 years,			0.40				
Yes	72 (31.4%)	114.09 ± 15.79		30.47 ± 4.85	36.29 ± 8.33	32.47 ± 4.08	14.86 ± 5.38
No	157 (68.6%)	112.07 ± 17.56		30.17 ± 5.53	35.29 ± 8.98	31.89 ± 4.79	14.71 ± 5.19
Total	229 (100%)	112.71 ± 17.02		30.26 ± 5.32	35.61 ± 8.77	32.07 ± 4.58	14.75 ± 5.24

* *p* < 0.05, SD: standard derivation.

**Table 2:** The results of CVI on VAI using the Davis technique.

	Expert Opinion (*n* = 15)				
	Appropriate	Need Minor Revision	Need Major Revision	Not Appropriate	CVI
Item1	15	0	0	0	1.00
Item2	14	1	0	0	1.00
Item3	15	0	0	0	1.00
Item4	15	0	0	0	1.00
Item5	11	3	1	0	0.93
Item6	13	2	0	0	1.00
Item7	15	0	0	0	1.00
Item8	12	3	0	0	1.00
Item9	13	2	0	0	1.00
Item10	9	5	1	0	0.93
Item11	10	5	0	0	1.00
Item12	14	1	0	0	1.00
Item13	10	3	2	0	0.86
Item14	9	4	2	0	0.86
Item15	15	0	0	0	1.00
Item16	8	5	2	0	0.86
Item17	12	2	1	0	0.93
Item18	12	2	1	0	0.93
Item19	12	2	1	0	0.93
Item20	11	3	1	0	0.93

CVI: Content Validity Index VAI: Vaccine Acceptance Instrument.

**Table 3:** Exploratory factor analysis * of the Vaccine Acceptance Instrument.

Items	Factor 1	Factor 2	Factor 3	Factor 4
I10	0.766			
I8	0.685			
I7	0.661			
I9	0.657			
I1	0.506			
I3 (R)		0.765		
I2 (R)		0.662		
I6 (R)		0.639		
I5 (R)		0.613		
I4 (R)		0.587		
I12 (R)		0.499		
I11 (R)		0.325		
I16 (R)			0.735	
I13 (R)			0.720	
I14 (R)			0.598	
I15			0.587	
I19			0.464	
I17 (R)				0.816
I18 (R)				0.712
I20				0.711
Eigenvalues	5.144	2.344	1.509	1.413
% of variance	25.57	11.72	7.54	7.06
Total variance explained, %	51.9			
Kaiser-Meyer-Olkin Measure of Sampling Adequacy	0.787			
Bartlett’s Test of Sphericity	1358.6			
df	190			
*p* value	<0.01			

* Extraction Method: Principal Component Analysis. Rotation Method: Promax with Kaiser Normalization. Rotation converged in 8 iterations. Items 2, 3, 4, 5, 6, 11, 12, 13, 14, 16, 17, and 18 are reversed items (R). df: Degrees of freedom.

**Table 4:** Confirmatory Factor Analysis fit indices of VAI and acceptable limits [31].

Fit Indices	The Scale Value	Reference Values for Acceptable Fit	Reference Values for Good Fit
X^2^/df	380.04/164 = 2.317	2 < X^2^/df ≤ 3	0 ≤ X^2^/df ≤ 2
RMSEA	0.076	0.05 < RMSEA ≤ 0.08	0 < RMSEA ≤ 0.05
GFI	0.90	0.90 ≤ GFI < 0.95	0.95 ≤ GFI < 1.0
AGFI	0.906	0.90 ≤ AGFI < 0.95	0.95 ≤ AGFI < 1.0
CFI	0.910	0.90 ≤ CFI < 0.95	0.95 ≤ CFI < 1.0
NFI	0.90	0.90 ≤ NFI < 0.95	0.95 ≤ NFI < 1.0

X^2^: chi-square; df: degrees of freedom, RMSEA: root-mean-square error of approximation, GFI: goodness-of-fit index, AGFI: adjusted goodness-of-fit index, CFI: comparative fit index, NFI: normed fit index.

**Table 5:** Results of item and reliability analyses of the VAI.

	Item Number	Mean	Standard Deviation	Item-Total Correlation	Cronbach’s Alpha If Item Deleted	Cronbach’s Alpha of the Subscale	ICC * of Test–Retest Analyses (95%CI **)
Factor 1	1	6.2	1.43	0.398	0.817	0.754	
	7	6.25	1.38	0.384	0.817		
	8	5.86	1.62	0.38	0.817		0.977 (0.96–0.987)
	9	5.76	1.60	0.432	0.815		
	10	6.17	1.43	0.408	0.816		
Factor 2	2	4.69	1.79	0.389	0.817	0.753	
	3	5.14	1.88	0.505	0.811		
	4	6.06	1.45	0.566	0.810		
	5	5.58	1.87	0.567	0.807		0.986 (0.976–0.992)
	6	4.48	2.24	0.241	0.827		
	11	4.29	2.45	0.321	0.823		
	12	5.34	1.95	0.614	0.804		
Factor 3	13	6.49	1.35	0.419	0.816	0.714	
	14	6.04	1.63	0.447	0.814		
	15	6.42	1.28	0.371	0.818		0.972 (0.951–0.984)
	16	6.69	1.08	0.386	0.818		
	19	6.41	1.28	0.308	0.820		
Factor 4	17	5.06	2.28	0.385	0.818	0.662	
	18	4.36	2.45	0.331	0.822		0.945 (0.903–0.969)
	20	5.32	2.02	0.331	0.820		
Total							0.992 (0.987–0.996)

* ICC: Intraclass Correlation Coefficient; ** CI: confidence interval.

## Data Availability

The anonymized dataset is available from the corresponding author upon reasonable request.
